# Behavioral Strategies of Phorid Parasitoids and Responses of Their Hosts, the Leaf-Cutting Ants

**DOI:** 10.1673/031.012.13501

**Published:** 2012-11-21

**Authors:** Luciana Elizalde, Patricia Julia Folgarait

**Affiliations:** ^1^ Laboratorio de Hormigas, Departamento de Ciencia y Tecnología, Universidad Nacional de Quilmes, Buenos Aires, Argentina; ^2^ Laboratorio Ecotono, Centro Regional Universitario Bariloche, Universidad Nacional del Comahue, Bariloche, Argentina

## Abstract

Host-searching and oviposition behaviors of parasitoids, and defensive responses of the hosts, are fundamental in shaping the ecology of host-parasitoid interactions. In order to uncover key behavioral features for the little known interactions between phorid parasitoids (Diptera: Phoridae) and their leaf-cutting ant hosts (Formicidae: Attini), host-related behavioral strategies (i.e., host searching and oviposition) for 13 phorid species, and host defensive responses (i.e., hitchhikers and particular body postures) for 11 ant species, were studied. Data was collected at 14 localities, one of them characterized by its high species richness for this host-parasitoid system. Phorid species showed both great variation and specificity in attacking behaviors. Some chose their hosts using either an ambush or an actively searching strategy, while some species attacked ants on different body parts, and specialized on ants performing different tasks, such as when ants were foraging, removing wastes to refuse piles, or repairing the nest. Combining all the behaviors recorded, most phorid species differed in performance in at least one, making it possible to recognize species in the field through their behavior. Phorid species that attacked hosts with greater activity levels showed overall higher attack rates, although there was no significant correlation between attack rates by most phorid species and ant activity outside the nest while parasitoids were attacking. The presence of phorids was a significant determinant for the presence of defensive behaviors by the ants. Although ant species varied in the incidence levels of these defensive behaviors, most ant species reacted against different phorids by utilizing similar behaviors, in contrast to what parasitoids do. General features of the observed phorid-ant interactions were parasitoid specialization and corresponding high interspecific variation in their behaviors, while their hosts showed generalized responses to attacks with high intraspecific variation. Behavioral patterns as well as specific features of these ant-parasitoid interactions are described, and their ecological importance discussed.

## Introduction

Any successful endoparasitoid must overcome a hierarchical set of barriers in order to oviposit in a host. The parasitoid must locate and encounter its prey, and, upon doing so, manage to insert an egg while overcoming the host's physical and behavioral defenses (Vinson 1976; Godfray 1994). Several aspects of the behavior of parasitoids can affect the response of the hosts, making the defensive response of the hosts more or less effective in preventing the parasitoid from leaving offspring. In general, behavioral mechanisms of hosts against parasites are the first line of defense (Kenneth 2005). For dipteran parasitoids with mobile adult hosts, behavioral defenses are a critical element to overcome, and one that effectively determines the host range of these flies (Feener and Brown 1997).

Leaf-cutting ants (Hymenoptera: Formicidae: Attini: *Acromyrmex* and *Atta*) cut plant tissue from surrounding vegetation and carry the pieces back to their nest using a persistent trail network (Kost et al. 2005). Inside the nest, the plant fragments are used as substrate for a symbiotic fungus they cultivate in underground chambers (Hölldobler and Wilson 1990). The fungus consumes part of the plant tissues, and the ants discard the rest (Hölldobler and Wilson 1990; Waddington and Hughes 2010). Some ant species deposit the refuse in special chambers inside the nest, but others carry the debris to refuse piles outside the nest (Hölldobler and Wilson 1990; Ballari et al. 2007). When ant workers are outside the nest for any reason, they could be attacked by phorid (Diptera: Phoridae) endoparasitoids, which use a piercing ovipositor to insert an egg inside the worker's body. The phorid species that *use Atta* as hosts do not use *Acromyrmex* ants (Elizalde and Folgarait 2011). The host-related behaviors (i.e., host searching and oviposition behaviors) of just a few species of phorid parasitoids of *Atta* are known (Feener and Moss 1990; Feener and Brown 1993; Tonhasca 1996; Erthal and Tonhasca 2000; Tonhasca et al. 2001; Bragança et al. 2002; Bragança and Medeiros 2006), but descriptions of behavioral patterns for parasitoids of *Acromyrmex* are nonexistent (but see Brown 1997). Moreover, no generalizations have been made for any of these parasitoids in terms of host-related behaviors.

Phorid species that frequently parasitize leafcutting ants belong mainly to three genera: *Apocephalus* (Brown 1997), *Eibesfeldtphora* (recently raised to the genus status, being previously a subgenus of *Neodohrniphora*, Disney et al. 2009), and *Myrmosicarius* (Disney et al. 2006). These parasitoids vary in their behaviors, such as ovipositing workers while transporting leaves in the foraging trail or while hosts are cutting the leaf fragment (Tonhasca 1996; Erthal and Tonhasca 2000; Bragança et al. 2002; Bragança and Medeiros 2006; Bragança et al. 2009), ovipositing using the load transported by the ant as a platform (Feener and Moss 1990) or not (Tonhasca 1996; Tonhasca et al. 2001; Bragança et al. 2009), and ovipositing in different parts of the ant host's body (Feener and Brown 1993; Tonhasca et al. 2001). Differences in the behavioral repertoire of the parasitoids reflect intrinsic properties of the species, or may depend on their hosts’ characteristics, such as host activity outside the nest. For example, one *Eibesfeldtphora* species attacked more often when ant foraging activity was higher (Tonhasca 1996). The oviposition rate is an important aspect of the behavior of phorids because it reflects the fitness of phorids and/or the behavioral effect they exert on their host (Orr et al. 1997). However, little is known about oviposition rates of these phorids (Tonhasca 1996; Bragança et al. 2009).

Phorids that parasitize leaf-cutting ants affect the foraging activity of their hosts through behavioral modifications (Tonhasca 1996; Bragança et al. 1998). The response behaviors of *Atta* ants against phorids include dropping their load (Tonhasca 1996); retreating to the nest (Orr 1992); moving legs, antennae, and mandibles (Eibl-Eibesfeldt 1967; Feener and Moss 1990); outrunning the phorid (Feener and Brown 1993); or adopting particular body postures in order to avoid oviposition (Tonhasca 1996; Bragança et al. 2002). Some of these behaviors can involve ants that are not directly at risk of being parasitized by phorids, in which case they constitute colony level responses against the parasitoids. One of these colony level behaviors against the phorids, and unique to leaf-cutting ants, is the presence of hitchhikers. Hitchhikers are small workers, too small to host the phorids, that ride on the leaves transported by bigger and suitable host workers (Eibl-Eibesfeldt 1967; Feener and Moss 1990). Through experimental and detailed observations in the field, Feener and Moss (1990) showed that hitchhikers had a defensive function against phorids that land on leaves to oviposit. Almost nothing is known about the behaviors that *Acromyrmex* ant species exhibit against the phorids that attack them, except for some occasional observations (Brown 1999).

In this work, the behavioral strategies used by different phorid species to oviposit on their leaf-cutting ant hosts, and their hosts’ behavioral responses to phorid attack, were described and compiled. Specifically, the following aspects of parasitoid behaviors were addressed: (1) establishing the generalities of host-related behaviors of phorids attacking *Acromyrmex* and *Atta* ants, (2) determining the existence of inter-specific differences in host attack rate and the time allocated by phorids to perform host-related behaviors, and (3) whether differences in attack rates within and among phorid species were related to the number of ant workers outside the nest. Then, focusing on host behaviors, the following characteristics were evaluated: (4) whether there was an association of hitchhiking and defensive body postures of the ants with the presence of phorids, and (5) the differences among host species in the behavioral defenses displayed against attacking phorid species.

## Materials and Methods

### Host-related behaviors of phorids

In order to describe host-related behaviors of phorids attacking leaf-cutting ants, the following were considered: (a) sites for host selection, (b) sites of the host body where oviposition occurs, (c) whether the fly landed on the ant or the load being carried by the ant to oviposit, and (d) the phorid's searching behavior for individual hosts. Data was collected in 12 localities in Argentina and two in Paraguay (sampling sites in Elizalde and Folgarait 2010). At each locality, and in at least three nests of each leaf-cutting ant species present, phorids were searched for in the sites where they are known to oviposit, such as foraging trails and cutting sites. In addition, phorids were searched for at external refuse piles, where some ant species dispose of their wastes (i.e., dead ants and the exhausted vegetal material used by the fungus). Once a phorid was found, its behavior was observed focusing on items (b), (c), and (d), as mentioned above. Observations of phorids from a very short distance by a trained, naked eye were more efficient for behavioral data gathering than using a video camera because video-recording in the field at the speed and distances these small phorids move was not possible. After completing the behavioral observation, phorids were captured in order to identify them to species. For completeness, unpublished data about the host-related behavior of a phorid species collected in La Selva Biological Station in Costa Rica were included. The behavioral observations were all performed during daylight hours and when ant activity was well established.

To compare differences among phorid species in time allocation to relevant aspects of their behavioral repertoire, detailed observations were performed in San Cristóbal (Santa Fe province, Argentina, 30° 12′ S, 61° 09′ W), the community with the greatest phorid richness known to date (Elizalde and Folgarait 2011). In this locality, the leaf-cutting ants present were *Atta vollenweideri* Forel and seven species of *Acromyrmex* (*Ac. crassispinus* Forel, *Ac. fracticornis* Forel, *Ac. heyeri* Forel, *Ac. hispidus* Santschi, *Ac. lobicornis* Emery, *Ac. lundii* Guerin-Meneville, and *Ac. striatus* Roger). Based on previous observations, and on those of Feener and Brown (1993), four host-related behaviors displayed by phorids were defined, and were the main activities performed: (1) perching: when the phorid was sitting on the side of the foraging trail, nest, or refuse pile, with the head directed towards the ants; (2) flying: when the phorid was flying to get close to an ant, came back to the perching site, went from perch to perch, or moved along the foraging trail; (3) attacking: when the phorid touched an ant for at least one second, so as to deposit an egg (as defined previously for other phorids, Tonhasca et al. 2001; Silva et al. 2008; Bragança et al. 2009) (this behavior is not referred to as oviposition because we did not confirm the presence of eggs); (4) landing on a leaf: when the phorid landed on the leaf transported by an ant, usually prior to an attack.

When a phorid female was found attacking a host, her behavior was described verbally by the observer and registered using an audiocassette recorder until at least three oviposition attempts were made on different workers. Recording observations in audiocassettes allowed quantifying the duration of each behavior. Following the observation period, the phorid was captured, and kept in a vial with alcohol, which was adequately labeled for later identification and to associate the phorid with the registered behaviors. The behavior of phorids while attacking ants was quantified for a total of 20 hours, for eight different species. Individual parasitoids were observed for nine minutes on average (SE = 0.7 min.).

Differences among phorid species in the percentage of time allocated to each of the mentioned behaviors was compared with Kruskal-Wallis analyses of variance since the data could not be normalized for most behaviors, even after transformations. The “landing on a leaf” behavior was not included, since it was performed by two species only, and just for a few seconds before ovipositing. Differences among phorid species behaviors were determined using non-parametric tests, and P-levels were adjusted using Holm's correction (Quinn and Keough 2002). Some phorid species use more than one host, but we only collected detailed behavioral data on the preferred host, because on the other hosts it was difficult to sample enough as to be used for statistical analyses. Behaviors for phorid species attacking *Acromyrmex* or *Atta* were compared separately because *Atta* had higher activity outside the nest than *Acromyrmex* (see Results), which may affect the time that phorid species allocate to each behavior, and also because they did not share hosts (Elizalde and Folgarait 2011). To compare foraging host activity among ant species, measurements of ant activity obtained in periods when no parasitoids were attacking were used, as to exclude any effect that phorids may exert on ant activity. For each ant species, at least 21 ant activity measurements were obtained for a wide range of temperatures (10–40° C) and only for adult nests. An ANOVA was performed (ant activity data was log-transformed to meet the assumptions of the test), and then a Tukey multiple comparisons test was used to find differences in foraging activity among ant species.

The attack rate for each parasitoid species in the high-diversity site was estimated as the number of attacking bouts that phorids performed per minute. Because the attack rate could depend on ant activity in foraging trails, a regression analysis was carried out between the number of ants per minute and attack rate. Ants per minute were estimated by averaging the number of ants returning to the nest during one minute counted before and after recording the observations of phorid behavior. Attack rates and ant activity were log-transformed to meet the assumptions of the analysis. Then, the residuals of the regression were used in a one-way ANOVA with parasitoid species as factor. In addition, to test for an effect of ant activity on phorid attack rates within species, non-parametric correlations between attack rates and average ant activity recorded during the same observation period were carried out.

The activity of ants outside the nest can be regarded as a feature at the species level, since some species of leaf-cutting ants had more foragers outside the nest than others (Hölldobler and Wilson 1990; Elizalde and Folgarait 2010; see Results), and therefore may affect the rate of parasitoid attack. To assess this hypothesis, and test the effect of variation of ant activity among phorid species, a correlation was carried out between the mean attack rate of parasitoid species and the mean ant activity at species level. The activity of each ant species was the average activity recorded for all sampling periods when no parasitoids were attacking, as to exclude any effect that phorids may exert on ant activity.

### Ant behaviors in response to phorids

To evaluate the association between the presence of parasitoids and the ants’ responses typically exhibited against phorids (i.e., hitchhiking and body postures), their incidence was recorded in three nests of each ant species present in the same 14 localities mentioned above. This sampling yielded information for more ant species than sampling at only one locality would have. The presence of ant body postures and hitchhikers was recorded while crawling slowly along the main part of a foraging trail for 30 minutes. All phorids observed during these 30-minute periods were collected in order to later identify them to species. Thirty-minute samplings for ant defense behaviors and phorid presence were also carried out at refuse piles, external to the nest, when present.

The relationships between the dependent variable (presence of hitchhikers or presence of defensive postures) and the independent variables (presence of phorids and ant species) were established using two logistic models (also called logit models in this case because dependent variables were categorical, Agresti 2002). These models give better estimations of ant defenses against phorids as an overall feature of the interaction than the proportions obtained directly from the sampled data because these proportions are smoothed by using the information from all the observations, not just the information involved in the proportion being considered (Agresti 2002). Log-likelihood ratio tests were used to evaluate the importance of the independent variables in the model. For these analyses, ant species in which defensive behaviors were not observed (*Acromyrmex balzani* Emery, *Ac. fracticornis*, and *Ac. rugosus* Smith) and *Ac. striatus*, which showed defensive behaviors at a very low frequency, were not included. In addition, *Ac. hispidus* was not included in the model accounting for hitchhiker presence because almost all their phorids were attacking in refuse piles, where no hitchhikers were registered (see Results). Data for each ant species were pooled across localities.

While recording focal observations of phorid behaviors in the high-diversity site, ants’ responses to phorids were registered in order to describe them and to evaluate whether ant species differed in their defenses according to phorid species attacking. Based on the repertoire of responses reported for *Atta* ants in reaction to phorids (Eibl-Eibesfeldt and Eibl-Eibesfeldt 1967; Feener and Moss 1990; Feener and Brown 1993; Tonhasca 1996; Bragança et al. 2002; Bragança et al. 2009), the behaviors described for *Solenopsis* ants in response to their phorid parasitoids (Wuellner et al. 2002), and our observations, several behaviors of ants in response to phorids were defined. The percentage that each behavior was performed by each ant species was calculated, discriminated by the phorid species attacking. Since phorids, the focus of the observations, flew very fast, it was not possible to record the reactions of all ants.

**Figure 1.**
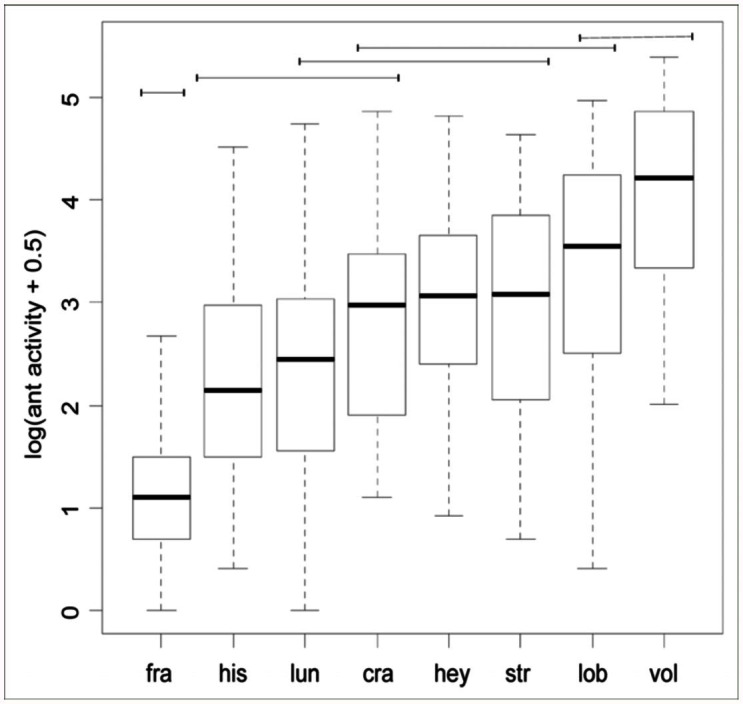
Box-whisker plot for ant foraging activity from eight leaf-cutting ant species sampled in a high-richness locality. Ant names are abbreviated with the first three letters of the specific epithet: *Acromyrmex fracticornis, Acromyrmex hispidus, Acromyrmex lundii, Acromyrmex crassispinus, Acromyrmex heyeri, Acromyrmex striatus, Acromyrmex lobicornis*, and *Atta vollenweideri*). Lines group ant species with similar values of activity (*P* > 0.05, Tukey multiple comparison test). High quality figures are available online.

The responses of ants against phorid attacks included: (1) not reacting, (2) outrunning the pursuing phorid (the change in velocity was very obvious, although it was not measured), or (3) thrashing legs and antennae trying to dislodge the phorid. Additionally, the responses included (4) adopting one of the following body postures, for which ants generally stopped walking and sometimes dropped the load: (a) ball: the ant curled on itself, forming a small ball, with the head down and the gaster between the legs; (b) biting: the head was directed upwards, antennae and legs were stretched out, and the mandibles were open upwards; (c) C posture: the gaster was located between the legs, forming a “C” with the body (see Figure 1c in Eibl-Eibesfeldt and Eibl-Eibesfeldt 1967) (the biting and C postures were very similar, and although they were recorded separately, in some instances an ant would switch from one to the other); (d) gaster down: the gaster was directed towards the ground in a way that the tip was not accessible (the gaster was not located between the legs, and the ant was able to walk while adopting this posture); (e) head against thorax: the ant put its head against the thorax, impeding the access to the neck area; (f) laying: the ant lay down on its side or crouched against the ground.

These postures were generally adopted by the ants at the moment the phorids touched them, and were called “post-attack responses”. But in some instances, an ant could adopt the posture when the phorid was near, and was thus a “pre-attack response”. Colony level responses of ants to phorids (i.e., ants showing a reaction although they were not being pursued by phorids) included the biting or C postures. Usually these ants were near the attacked ant or detected the phorid that was flying past them. Another colony level response, called “tending,“ occurred when 5– 15 nest-mates approached an attacked ant in a defensive posture and touched the attacked ant with their mandibles and antennae (Feener and Moss 1990; Tonhasca et al. 2001).

Ant species were identified in the field and corroborated later in the laboratory using the available keys (Bonetto 1959; Gonçalves 1961). Phorids could not be identified in the field, and were collected and identified in the laboratory using keys (Brown 1997; Disney et al. 2006; Disney et al. 2008; Disney et al. 2009; Brown et al. 2010). Voucher specimens of phorids and ants were deposited in Museo Bernardino Rivadavia (Buenos Aires, Argentina). Statistical analyses were performed in the R environment (R Development Core Team 2010).

## Results

### Host-related behaviors of phorids

The generalities of host-related behaviors of phorids attacking *Acromyrmex* and *Atta* ants were described by collecting data from ant nests at 14 localities. In addition, from a high-species richness locality, the existence of inter-specific differences in host attack rate, and the time allocated to perform host-related behaviors, were evaluated, as well as the possible effect of the number of ant workers outside the nest on attack rates within and among phorid species.

Phorids belonging to the same species showed the same host-related behaviors, such as site of egg insertion, landing on host body or load to oviposit, and strategy to select individual hosts even when attacking different host species, in all localities sampled. Most phorid species searched for their leaf-cutting ant hosts in foraging trails (Table 1), and several phorid species were reported attacking ants when taking waste out of the nest and working on the external refuse piles (Table 1). One of these parasitoids, *Myrmosicarius longipalpis* Disney, was exclusively found attacking ants at the refuse piles of its host *Ac. hispidus*, whereas *Myrmosicarius crudelis* Borgmeier and *Myrmosicarius gracilipes* Borgmeier were found at higher abundance at refuse piles, since only 28% (out of a total 88 individuals) and 39% (of 18) of the individuals were found in foraging trails of *Ac. crassispinus*, respectively. On the other hand, *Apocephalus neivai* Borgmeier and *Myrmosicarius cristobalensis* Disney were found mostly on foraging trails, with only 4% of the individuals (of 141) and 11% (of 97) attacking ants at refuse piles of *Ac. crassispinus* and *Ac. lobicornis*, respectively. In addition, *Myrmosicarius catharinensis* Borgmeier occasionally (4% of 115 individuals) attacked ants that were performing nest maintenance tasks (i.e., arranging sticks). Since phorids from the same locality were attacking the same host species on trails and refuse piles, or while they were maintaining the nest, this behavioral variation was not related to having pooled data across different host species or sampling localities.

**Table 1.**
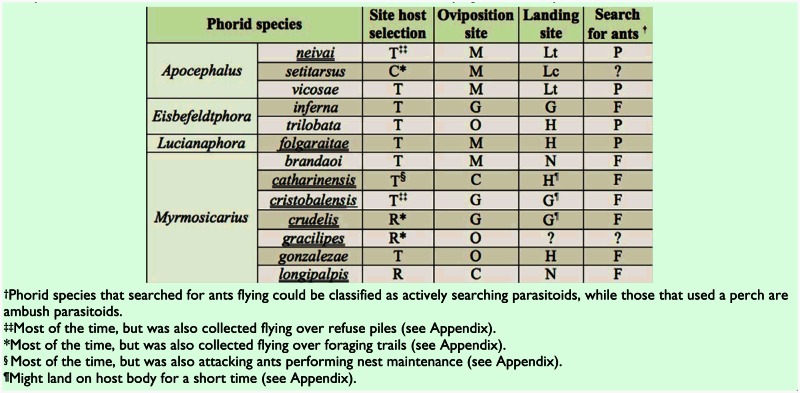
Summary of main host-related phorid parasitoid behaviors (for more details see Appendix). Phorids that attack *Acromyrmex* host species are underlined. Abbreviations: ? = no information gathered; for “Site for host selection” column: C = cutting site, R = refuse pile, T = foraging trail; for “Oviposition site” column: C = clypeus area, G = tip of gaster, M = mandible or maxillae insertion, O = occiput or near; for “Landing site” column: G = gaster, H = head, Lc = leaf being cut, Lt = leaf transported, N = does not land; for “Search for ants” column: F = while flying, P = from a perch.

The site of oviposition on the host's body varied across, but not within, phorid species (Table 1). Most species used different areas of the ants’ heads to oviposit (in the front, back, mouth area; see Appendix), and *Apocephalus* species oviposited exclusively in the head (Table 1). However, some species belonging to *Eibesfeldtphora* and *Myrmosicarius* also oviposited in the gaster.

Phorid species could be classified according to how they searched for hosts. Some species searched for their hosts mostly from a perch (Table 1). These phorids started to fly, following a particular host, and then usually returned to the perch. These species, such as *Ap. neivai* among the species that attack *Acromyrmex*, and *Eibesfeldtphora trilobata* Disney among the species that attack *Atta*, employed the ambush strategy (see Barrows 2001). Other species used an actively searching strategy (see Barrows 2001), mainly choosing their hosts from the air (Table 1). Examples using this strategy included *Myrmosicarius brandaoi* Disney and *M. gonzalezae* Disney among species that attack *Atta*, and *M. catharinensis* Borgmeier and *M. longipalpis* among species that use *Acromyrmex* as hosts (Table 1). All species in the genera *Apocephalus* known to date are mainly ambush parasitoids, while all the *Myrmosicarius* species use the actively searching strategy (Table 1, Appendix). In the case of *Eibesfeldtphora* species, *E. inferna* behaved as an actively searching parasitoid, while *E. trilobata* used an ambush behavior; however other species of this genus reported in the literature (*E. curvinervis* and *E. tonhascai;* Feener and Brown 1993; Tonhasca 1996) have shown to be ambush parasitoids.

Detailed behavioral observations of a group of phorid species in the high-diversity site supported the above dichotomy found in searching strategies. Phorids that searched their host from a perch, like *E. trilobata* and *Ap. neivai*, spent more time perching than other phorids (65 and 95% of the time, respectively, Table 2; H = 15.9, df = 2, *p* < 0.01 for perching behavior in *Atta*'*s* phorids; H = 15.7, df = 4, *p* < 0.01 for *Acromyrmex*'s phorids). Meanwhile, *Myrmosicarius* species, using an actively searching strategy, were flying for a higher percentage of time compared to species using the ambush strategy (Table 2; H = 6.1, df = 2, *p* = 0.05 for flying behavior in phorid parasitoids of *Atta;* H = 20.6, df = 4, *p* < 0.01 for phorid parasitoids of *Acromyrmex*). Two *Myrmosicarius* species (*M. cristobalensis* and *M. crudelis*) spent, however, higher percentages of their time compared to species using the ambush strategy (Table 2; H = 6.1, df = 2, *p* = 0.05 for flying behavior in phorid parasitoids of *Atta*; H = 20.6, df = 4, *p* < 0.01 for phorid parasitoids of *Acromyrmex*). Two *Myrmosicarius* species (*M. cristobalensis* and *M. crudelis*) spent, however, higher percentages of their time perching than flying (Table 2). These results were possibly due to these phorids resting more than the other species. On the other hand, some species showed differences in the time spent attacking ants, with *M. gonzalezae* being the species that spent a greater proportion of the time attacking (almost 15% of total time, Table 2; H = 14.9, df = 2, *p* *<* 0.01 for attacking behavior in *Atta* phorids), whereas the other species only spent 1–3% of the time performing this behavior (Table 2). Phorid parasitoids of *Acromyrmex* differed in the time spent attacking as well (H = 12.0, d.f = 4, *p* = 0.02), with *M. longipalpis* spending less time attacking than *Ap. neivai* and *M. crudelis* (Table 2). Because *At. vollenweideri* had higher activity outside the nest than most *Acromyrmex* species (F7,274 = 24.5, *p* < 0.001; Figure 1), and that difference may affect the time that phorids attacking each host allocate to different behaviors as well as attack rates, phorid species attacking each genera were compared separately.

**Table 2.**
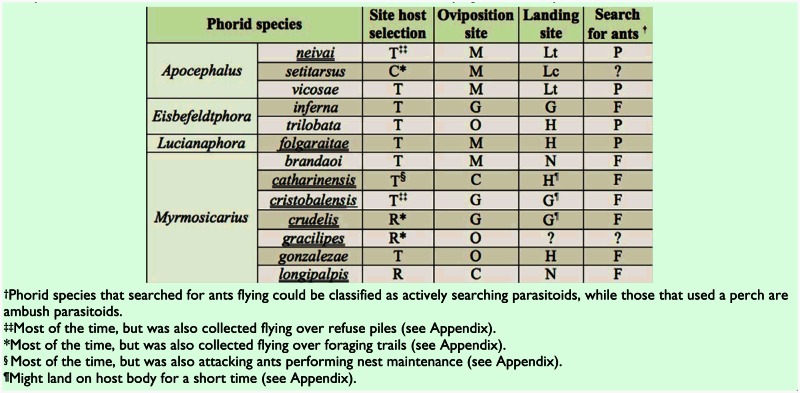
Percentage of the time (median, first, and third quartiles between parentheses) that each phorid species spent performing host-related behaviors: attacking, perching, and flying; and mean attack rates per minute (standard deviation between parentheses). a) Phorid species attacking foragers of *Atta vollenweideri*. b) Phorid species that attack *Acromyrmex* hosts, first indicating the host species on which data was collected, and the task that ants were performing between parentheses (f = foraging, w = waste removal).

There were no differences in attack rates among phorid species attacking *Atta* (F_2,54_ = 2.14,*p* = 0.13; Table 2) or *Acromyrmex* (F_4,39_ = 1.45, *p* = 0.23; Table 2). Only for *M*. *gonzalezae* and *M. crudelis* was there a significant and positive correlation between phorid attack rate and ant activity (rho = 0.48, *p* = 0.03, N = 16; rho = 0.60,*p* = 0.01, N = 13, respectively), and no correlation was found in any of the other species tested (*M. brandaoi*: rho = 0.32, *p* = 0.20, N = 9; *M. catharinensis*: rho = -0.16, *p* = 0.63, N = 7; *M. cristobalensis:* rho = -0.18, *p* = 0.72, N = 13; *E. trilobata:* rho = 0.08, *p* = 0.32, N = 32). However, mean ant activity measured as a species trait (Figure 1) showed a positive correlation with mean attack rates per parasitoid species (rho = 0.84,*p* = 0.009, N = 7).

A complete description of the behavior of each phorid species and the defensive behaviors of the ants is given in the Appendix.

### Ant behaviors in response to phorids

The association of hitchhiking and defensive body postures of 11 ant species with the presence/absence of phorids was tested using logistic models. Furthermore, differences among host species in their behavioral defenses displayed against phorid species were described and compared in the high-richness locality.

Hitchhikers were recorded in 36% of the 30-minute samplings and defensive body postures in 37% of them (577 total sampling periods of 30 minutes, where *Atta saltensis* was the species less sampled (with 34 sampling periods), and *Ac. lundii* the species most sampled (with 145 periods)). However, since phorids were not frequently found in most ant nests (only 26% of the nests sampled for 30 minutes had phorids), these percentages increased to 51% in periods with hitchhikers and to 69% in periods with defense postures of the ants when only considering the sampling periods with phorids’ presence.

Hitchhikers were found in several ant species without previous records of this behavior: *At. saltensis* Forel, *At. vollenweideri, Acromyrmex ambiguus* Emery, *Ac. crassispinus, Ac. heyeri, Ac. hispidus, Ac. lobicornis, Ac. lundii*, and *Ac. striatus*. Only three *Acromyrmex* species had neither hitchhikers nor body postures: *Ac. fracticornis, Ac. rugosus*, and *Ac. balzani*. In fact, no phorids were collected attacking the last species, and phorids over *Ac. rugosus* were recorded in one opportunity only. No hitchhiking was recorded in waste remover workers, although they did display defensive postures. No more than three hitchhikers were recorded per leaf, the most frequent situation being only one.

**Figure 2.**
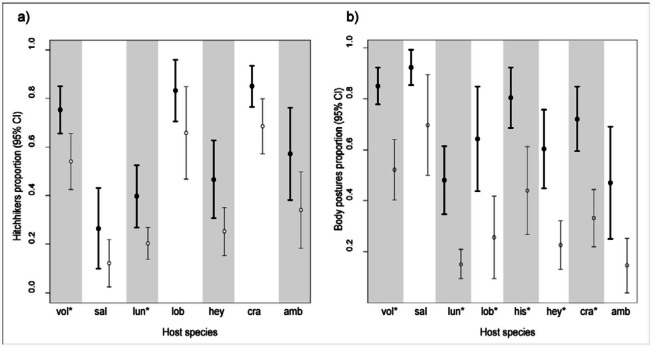
Estimated proportions (dots) and 95% confidence intervals (lines) from the logistic model for: a) hitchhiker presence, and b) ant body postures presence. Probabilities are discriminated by ant host species (x-axis, ant names abbreviated with the first three letters of the specific epithet: *Atta vollenweideri, Atta saltensis, Acromyrmex lundii, Acromyrmex lobicornis, Acromyrmex hispidus, Acromyrmex heyeri, Acromyrmex crassispinus, Acromyrmex ambiguus*); and phorid presence (full dots and bolded lines indicate phorid presence, absence shown with white dots). Asterisks next to species labels indicate the species that differed significantly according to Fisher's exact tests (see Results). High quality figures are available online.

Both the presence of phorids and the host species were important determinants of the probability for the presence of hitchhikers (phorid presence: deviance = 17.9, df = 1,*p* < 0.001; host species: deviance = 87.2, df = 6,*p* < 0.001; deviance of the logistic model = 2.08, df = 6,*p* = 0.91 indicates a good fit to the data (Agresti 2002); Figure 2a). Phorid presence (estimated coefficient from the model b = 0.95, SE = 0.24) increased the chances of finding hitchhikers by *e*^0.95^. It was thus 2.58 times more likely to find them during a 30-minute sampling period compared to periods without phorids (the adjusted mean probability of hitchhiker presence for each ant species was higher when phorids were present in comparison to absent, Figure 2a). Since confidence intervals overlapped considerably in some ant species, the proportion of samplings with hitchhikers when phorids were present or absent for each ant species were compared using Fisher's exact tests. Only *At. vollenweideri* and *Ac. lundii* showed a significant association between the presence of phorids and hitchhikers (*p* < 0.05). Meanwhile, for *Ac. ambiguus*, the association between these variables was nearly significant (*p* = 0.07).

The presence of hitchhikers was recorded when phorids that land on leaves were the only species of parasitoid ovipositing, and when other phorid species were attacking alone. Thus, to evaluate whether phorids that do not land on the leaves also triggered the hitchhiker defense, another logistic model was used, but excluding from the dataset sampling periods with phorid species landing on the leaf to oviposit (*Ap. neivai* and *Apocephalus vicosae* Disney; see Table 1). Similar results to those with the complete data set were obtained, with both phorid presence and ant species as important variables to account for hitchhiker presence (phorid presence: deviance = 10.8, df = 1*,p* < 0.01; host species: deviance = 82.7, df = 6,*p* < 0.01; deviance of the logistic model = 4.01, df = 6, *p* = 0.71), suggesting that hitchhikers may also have a defense function against phorids that do not land on the leaves.

Both phorid presence and ant species were important when accounting for occurrence of ant body postures according to a logistic model (phorid presence: deviance = 61.4, df = 1, *p* < 0.001; host species: deviance = 70.8, df = 7, *p* < 0.001; deviance of the logistic model = 8.87, df = 7 and *p* = 0.26 indicates a good fit to the data; Figure 2b). Phorid presence (estimated coefficient b= 1.65, SE = 0.24) increased the chances to find defensive postures by 5.2 times (*e*^1.65^). In addition, ant species showed different probabilities of displaying postures (Figure 2b).

Defensive behaviors of ants against phorids were similar for most ant species, irrespective of the phorid species attacking, although the proportion in which they were displayed differed (Table 3). *Atta* and *Acromyrmex* species showed the same body postures in response to phorids (Table 3, pooling species of each genus), except for the “thorax against head” and “gaster down” behaviors found only in *Atta* and in some *Acromyrmex*, respectively (but see Appendix for the last behavior elicited by an *Atta* parasitoid, *Eibesfeldtphora inferna* Brown). However, the percentages of display for each behavior differed according to which phorid species was attacking (Table 3). Most ants reacted against the phorids with the ball posture when they were touched by the parasitoids (Table 3). *At. vollenweideri* reacted to attacks by *E. trilobata* and *M. gonzalezae*, phorid species with different oviposition strategies, with similar postures, although varying in the percentage that each one was displayed (Table 3). Similar defensive reactions by the ants were found when comparing *Acromyrmex* species; six behaviors were shared by *Ac. lobicornis* foragers reacting against *M. cristobalensis* and by *Ac. crassispinus* waste removers against *M. crudelis* (pooling pre and post-attack behaviors; Table 3). Both phorid species attacked the gaster, and ants reacted against them by lowering it, even when the focal ants were foragers or waste removers.

**Table 3.**
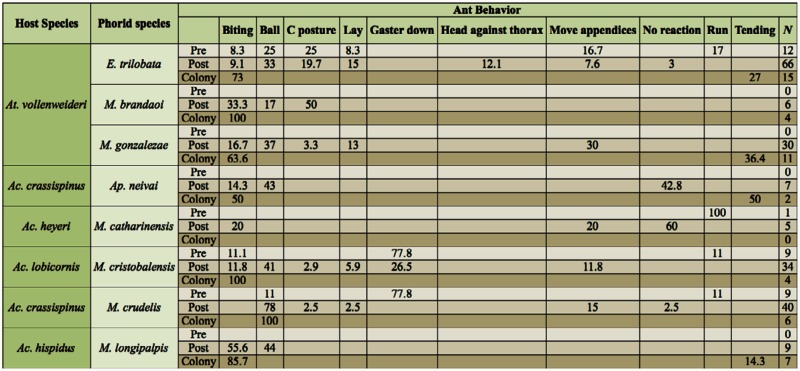
Percentages for the behavioral responses of ant species towards attacking phorid species, depending on whether the attack was displayed previously (Pre) or after (Post) the ant was touched by the phorid. In addition, colony responses by ants not pursued by phorids (but from the trail where an ant was attacked) are included. The number of ants for which behaviors were recorded is included for pre and post attack behaviors; for colony level responses ‘n’ represents the number of times that the behavior was recorded.

## Discussion

Parasitoids are under strong natural selection to evolve efficient host search and attack strategies because they depend on their hosts for their fitness. Furthermore, since parasitoids’ reproduction results in the death of their hosts, parasitoids may be important factors shaping host behaviors and morphology in order to be well defended (Price et al. 1988; Gross 1993; Nyman et al. 2007). This study shed light on some possible effects of parasitoids on hosts’ behavior, and on the great variety of host-related behaviors of phorid species that parasitize leaf-cutting ants, as well as on the diverse response behaviors that their hosts display against these parasitoids in natural field conditions. Field behavioral studies are particularly relevant because they allow us to find out the adaptations instead of speculating about hostparasitoids interaction possibilities, and also because behavior is often context dependent (Heimpel and Casas 2008).

The diversity of parasitoid behaviors evaluated in this study included species using an ambush strategy and species actively searching for hosts to oviposit. In addition, some phorids oviposited during flight, and others used the ant load, the leaf being cut or the ant's body, to land and then oviposit. These phorids attacked ants performing different tasks, such as foragers, cutters, waste removers, or workers doing nest maintenance. Furthermore, some parasitoids attacked different parts of the hosts’ anatomy, mainly different parts of the head or at the tip of the abdomen, approaching them from the front, the back, or the side. Most phorid species differed in at least one of the mentioned characteristics, making it possible to recognize the species in the field through their behavior. We were able to identify them by their particular behaviors even in the locality with the richest leaf-cutting ant phorid parasitoid guild ever reported (Elizalde and Folgarait 2011). Therefore, this work not only contributes significantly to the behavioral ecology field, but it indirectly contributes to the taxonomical one by using field characteristics that are easy to recognize by non-taxonomists. Furthermore, the greatest merit of this work relies on the study of *Acromyrmex* parasitoids, for which nothing was known previously.

In general, each phorid species specialized in attacking ants performing a particular activity outside the nest. The use of different sites to search for hosts suggests that different cues for host location may be used by different phorid species, although nothing is known about long distance host location by these phorids (for short distance cues, see Gazal et al. 2009). For the first time, the behaviors of phorid species attacking ants when performing nest maintenance and taking out wastes to refuse piles were described in detail. Given that ants at refuse piles are not in direct contact with the food supply (Ballari et al. 2007; Waddington and Hughes 2010) and are likely of less nutritional quality, one may wonder why phorids were using such ants as hosts. For example, *Ac. hispidus* was attacked by *M. longipalpis* in refuse piles only. In addition to the relatively low foraging activity levels of *Ac. hispidus* (Figure 1), there were several instances when waste removers were active but there were no foragers outside the nest (Elizalde, personal observation). In fact, *M. cristobalensis* used the refuse piles as a host-searching site more opportunistically, when there were no ants foraging in the trails (see Appendix). Thus, this higher availability of ants at refuse piles may exert a strong selection factor for phorids to specialize on waste removers.

A behavioral distinction between phorid species was the use of different parts of the ants’ bodies to lay eggs: a group of six species (including *E. bragancai*, Bragança et al. 2003; *E. delcinata*, Brown 2001; and *E. erthali*, Bragança et al. 2003) in two genera oviposited on the tip of the gaster; the other species oviposited on the head, near the mouth parts or neck. Because leaf-cutting ants have a hard exoskeleton, the oviposition sites were places of the ants’ bodies with little sclerotization. This was also observed for Euphorinae hymenopteran parasitoids, which use mainly adult beetles with a very hard exoskeleton as hosts, and oviposit in the mouth, the base of antennae, and the anus (Shaw and Illoy 2004). Segregation to oviposit on different body parts was reported in other phorid species sharing the same host species (Brown 1999). However, for phorids parasitizing leaf-cutting ants, this segregation does not avoid larval competition for food or space inside the host, at least for the great majority of species that use the head of the ant host to pupariate, and only have one individual develop per host (Elizalde and Folgarait 2011). Nevertheless, these differences among phorid species in hosts’ body sites for oviposition, as well as those mentioned above for host location sites (foraging trails, refuse piles, cutting sites), suggest a role for host niche segregation as a promoter of diversity in host-related behaviors of these parasitoids, deserving further study.

Differences found in host selection and attack behaviors may imply particular skills and represent different strategies used by the phorids to avoid host behavioral defenses. Phorids using an active searching strategy for host location, such as *Myrmosicarius* species, were very good flyers, were able to fly backwards, to hover in the air, and showed relatively high speed of flight (see Appendix).

Thus, these species spent most of their time flying, choosing the host during flight and landing briefly on the host's body to oviposit. In addition, they flew along the foraging trail for relatively long distances, which may have helped them to evade the colony level behavioral responses by several ants that stayed in biting or C postures in the area of the foraging trail where they detected a parasitoid. This colony level defense, however, seems more effective against phorids that used the ambush strategy because the phorids tended to return to the same perch, reusing the same area of the foraging trail where ants maintained the defensive postures elicited by previous attacks.

The ambush strategy was employed by most *Eibesfeldtphora* species (including *E. curvinervis*, Feener and Brown 1993; and *E. tonhascai*, Tonhasca 1996), taking advantage of the behavior of the ants that foraged along a trail. A key to understanding the benefits of using the ambush strategy may be the fact that these phorids used less abundant, big ants as hosts (Feener and Brown 1993; Tonhasca 1996; Bragança et al. 2002; Elizalde and Folgarait 2011). Since big leaf-cutting ants are present in low numbers in foraging trails (Hölldobler and Wilson 1990), waiting for these hosts to pass by under a perch, instead of flying along the foraging trail in search for them, represents a saving energy strategy. *E. inferna* was the only exception, because it used an actively searching strategy, possibly due to the fact that this species oviposits on the more abundant median ants (1.56 mm median head width of *At. cephalotes* (Elizalde, unpublished results), compared to 2.12 mm median head width for foragers of that host species attacked by *E. curvinervis* (Feener and Brown 1993)).

Avoiding hosts’ defensive behaviors is part of the oviposition strategy displayed by most *Apocephalus* species. On one hand, *Apocephalus setitarsus* (this study), *Ap. attophilus* (Erthal and Tonhasca 2000), and *Ap. wallerae* (Waller and Moser 1990) attacked ants while they were busy cutting leaves, a time consuming task (an *At. vollenweideri* worker takes between 10–30 minutes to cut a leaf (Röschard and Roces 2003)). Moreover, *At. vollenweideri* ants cutting grasses are too far apart from each other to elicit a colony level defense (but see Eibl-Eibesfeldt and Eibl-Eibesfeldt 1967 and Erthal and Tonhasca 2000 for minima workers defending cutters). However, if the ant somehow detected the presence of phorids, it stopped cutting the leaf and ran away (see Appendix). On the other hand, those *Apocephalus* phorids that landed on ants’ loads to oviposit, such as *Ap. neivai, Ap. vicosae*, and *Ap. setitarsus*, usually approached the ant by short flights or walking, possibly as a way to reduce the chance of detection by ants (Erthal and Tonhasca 2000).

Regardless if the attack results in an effective oviposition or not, attack rates should still be considered when assessing the indirect potential effects that phorids have on their hosts, i.e., through the modification of the behaviors that ants display when they detect the phorids. Attack rates varied much within the same species, and no significant differences were found among species. However, *M. gonzalezae* spent the most time attacking ants, most likely since it took them several seconds to oviposit (see Appendix). Thus, this species could negatively affect the ants more than other species that spend a shorter time attacking. It is possible that some of the variation found in attack rates within phorid species was related to changes in ant foraging activity, given that for some phorid species (*M. gonzalezae* and *M. crudelis*) there was a positive relationship between attack rate and ant foraging activity, similar to what was found for an *Eibesfeldtphora* parasitoid of *Atta* (Tonhasca 1996; but Bragança et al. 2009 did not find such relationship). Given a scenario where phorid attack rates increase as a consequence of ant activity and, in turn, ant activity decreases as a consequence of phorids attack rate, the lack of a simple relationship between those variables is not surprising. Notwithstanding, ant activity measured as a species trait seems to be an important variable shaping the behavior in this interaction, since phorid species with higher attack rates were parasitizing ant species with overall higher activity outside the nest.

Evidence was found supporting the defensive function of hitchhikers as a general feature against phorids (Eibl-Eibesfeldt 1967; Feener and Moss 1990; Linksvayer et al. 2002), even for phorid species that do not land on the leaves transported to oviposit, as was originally proposed (Feener and Moss 1990). The overall trend shown by the logistic model was that all ant species had a higher proportion of hitchhikers’ presence when phorids were attacking them, even if only *At. vollenweideri* and *Ac. lundii* exhibited a significant positive association of hitchhiker and phorid presence when tested independently. As the goal here was to find overall trends, data was gathered by recording the presence of ant defenses against phorids for extended periods of time to assess their association with parasitoids. This measurement seems more appropriate than using another measurement, such as the proportion of ants with hitchhikers or body postures during a sampling period, due to differences in the behavior of parasitoid species and variation in ant activity among species. In fact, a source of variation not included in these analyses may be related to different phorid species exerting differential influence on the hitchhiking response by the ants (Feener and Brown 1993). Data collected here was not adequate to test this idea, since not enough instances were obtained with phorid species attacking singly. Some ants, such as *Ac. lobicornis* and *Ac. crassispinus*, were highly likely to exhibit hitchhikers when no phorids were present. Although it is possible that the phorid that triggered the hitchhiking behavior was no longer present while sampling occurred, other hypotheses have been raised as to account for the function of this peculiar behavior. There is supporting data for hitchhikers as cleaners of leaves from potential pathogens of the fungus cultured during transportation, as well as for sap ingestion from the edges of the recently cut leaf (Vieira-Neto et al. 2006; Griffiths and Hughes 2010). In any case, the overall low incidence of hitchhikers found in the field for most ant species (around 36% of the sampling periods with hitchhikers), as well as the low proportion of time that minima ants spent hitchhiking (Griffiths and Hughes 2010), supports the idea that it is too costly to afford continuous hitchhiking, and it should be an adjustable behavior according to the needs of the colony (Feener and Moss 1990).

All *Atta* species sampled exhibited hitchhikers (Linksvayer et al. 2002; this study), and seven *Acromyrmex* species also had hitchhikers. Even grass-cutting species like *At. vollenweideri* and *Ac. heyeri* had hitchhikers. Only two species were reported without hitchhikers, *Ac. octospinosus* and *Ac. versicolor* (reviewed in Linksvayer et al. 2002), and three species, *Ac. balzani, Ac. rugosus*, and *Ac. fracticornis*, did not show hitchhiking during this study. Although *Ac. balzani* and *Ac. rugosus* were not sampled enough as to affirm that they entirely lack the hitchhiking behavior, *Ac. fracticornis* was sampled intensively during different times of a year, and for over 67 hours. In addition, *Ac. fracticornis, Ac. rugosus*, and *Ac. octospinosus* had very low incidence of phorids (for the first two species (Elizalde, personal observation); for *Ac. octospinosus* Brown 1999). Therefore, it is possible that this scarce presence of phorids did not exert enough pressure to allocate workers as hitchhikers in these *Acromyrmex* species with small colony sizes (Wetterer 1991; Soares et al. 2006), where the cost of that defense could be very high.

Ant body postures were more associated with phorids than hitchhikers for most ant species. In fact, ants in biting postures captured and killed phorid flies on several occasions (see Appendix), confirming that it was an effective defense against these parasitoids. Moreover, it was proposed that these postures could be used as an indication of phorids attacking *Atta sexdens* on foraging trails (Bragança et al. 1998). However, in a few cases, small non-parasitoid flies induced some of these postures in the ants. It is possible that the ants did not detect the difference between phorids and other small flies. This could explain the instances when there were ants with defensive postures but no phorids attacking. It is also possible, as proposed above for hitchhikers, that the phorid that had triggered the response flew away before sampling.

When several phorid species were present simultaneously, ant body postures did not seem to be specific against each phorid species, as was suggested previously when considering a few phorid species (Bragança et al. 2002). A body posture that was only observed against phorids that oviposited at the end of the gaster (*E. inferna* (see Appendix), *M. crudelis*, and *M. cristobalensis*) was lowering down the gaster. Since phorids that oviposit in the gaster follow the prospective host, approaching its rear part for some seconds before ovipositing, ants might be able to discriminate phorids by their approach behavior, and react accordingly. As suggested for phorids that parasitize *Solenopsis* (Porter et al. 1995), it is possible that ants may become aware of the area where a phorid will attack by sensing the wing buzzing in a particular sector. Similar postures, such as the C and ball, are common in *Solenopsis* ants when attacked by *Pseudacteon* phorids (Wuellner et al. 2002), suggesting that ant body postures against phorids are generalized, even across different ant tribes.

A general feature of this phorid-ant interaction seems to be the high interspecific variation found in phorid behaviors, even after considering the possible constraints imposed by evolution by which *Myrmosicarius* use mainly an actively searching strategy, and *Apocephalus* and *Eibesfeldtphora* are ambush parasitoids. Meanwhile, the hosts showed high intraspecific variation in their responses to these phorid parasitoids, although the responses were similar among ant species and genera. This supports, at least in part, the asymmetry hypothesis proposed for parasitoid-host interactions (Lapchin 2002; Lapchin and Guillemaud 2005), according to which the parasitoids evolve specialized behaviors as they are able to select which host to attack, but the hosts acquire generalized responses against different parasitoid species since they are not capable of predicting which parasitoid species will attack them.

## Appendix

### Detailed description of phorid behavior

***Apocephalus neivai*** (observations made over *Ac. crassispinus, Ac. heyeri, Ac. lobicornis*, and *Ac. lundii*, mostly in San Cristóbal, Santa Fe province, and Noetinger, Córdoba province, Argentina). This phorid was usually perched at the side of the foraging trail, with its head directed towards the foraging trail, occasionally flying from perch to perch. When an ant carrying a leaf passed under the perch, the phorid flew to the leaf and walked on it towards the ant's head. Then, it touched the back of the head with its fore legs and then turned around to insert the ovipositor, near the insertion of the mandible in the right or left side, using the leaf as a platform. However, in several instances the female left the ant immediately after landing on the leaf or after touching the ant's head with its fore legs, without probing it with its ovipositor. This species went flying from perch to load, and in few cases from load to load, selecting ants to oviposit. After attacking, the phorid walked back to the tip of the leaf, flying from there to the perch.

The attacked ant as well as those near it adopted, in general, ball or biting postures. Two phorids were captured by the ants and then dismembered.

***Apocephalus setitarsus*** (observations made over *At. saltensis* in La María, INTA, Santiago del Estero, and *At. vollenweideri* at San Cristóobal, Santa Fe province; and Mercedes, Corrientes province, Argentina). The behavior of this species was observed on few occasions, due to its low abundance. Most of the time, phorids of this species were ovipositing on *Atta* workers while they were cutting leaves at cutting sites. In a few instances, this species was collected on the foraging trail; however, no attacks were recorded there. This species approached the host by walking. Once over the ant, the female touched with its forelegs the back of the head and the area of mandible insertion. Then, the ovipositing female turned around and positioned its ovipositor in the area of insertion of the mandibles, and remained there for 30–50 seconds.

If the ant detected the phorid, she moved her legs and antennae touching the area where the phorid was located. These ants stopped cutting and abandoned the area. In these cases, the phorid left the ant. In one case, a phorid was captured by the ants.

***Apocephalus vicosae*** (observations made over *At. vollenweideri* in San Cristóbal, Santa Fe, Argentina). This phorid was attacking ants in the foraging trails using the transported leaf as a platform. The ovipositing female went from perch to perch in short flying bouts, and was very difficult to follow when flying from the perch to the ant's load. *Ap. vicosae* reached the ant's load by walking or by a short flight towards the distal tip of the leaves carried by the ants. Once there, the phorid walked towards the side of the ant head, positioning its body perpendicular to the main axis of the ant body. Then, it extended its ovipositor to reach the area near the clypeus.

The ants kept walking with the phorids over them but some stopped when the phorids landed on the leaf. To capture this phorid while ovipositing on an ant, it was necessary to collect the ant, as the phorid seemed to get stuck by the ovipositor inside the ant's body. It was not possible to collect it with the aspirator. In one case, a phorid was captured by the ants.

***Eibesfeldtphora inferna*** (observations made over *At. cephalotes* in La Selva Biological Station, Costa Rica). This species was found most often on *At. cephalotes* trails, but was also collected near nest entrances. To attack, the fly approached the ant from its front and then positioned herself behind the ant. The fly followed the ant closely for as long as 100 cm. The phorid oriented its head towards the head of the ant and put the ovipositor in the direction of the ant anus, touching it while flying or after landing for approximately a second on the gaster. The exact place of oviposition could not be determined, but it was near the end of the gaster, most likely the anus itself.

*E. inferna* usually attacked ants that were carrying leaves, but it also attacked ants without leaves. Two flies of this species were riding on leaves carried by ants. After attacking a host, or trying to attack several ants, a phorid would either land on the sides of the trail, using a dry leaf or stick as a perch, or continue flying, searching for another host. If the phorid landed on a perch, it began flying again after some time, apparently unrelated to the number or size of the ants passing by the perch. This species did not use a perch for detecting appropriate hosts, because it was always observed flying for a while before attacking ants.

It seems that the ants detected the fly when being pursued. Ants reacted in two ways to *E. inferna:* walking faster to outrun the phorid, or lowering their abdomens against the ground, so that the tips were not accessible to the phorid. Both of these reactions were effective in dissuading the fly from ovipositing. Some ants even dropped the leaf and started walking faster.

When the ants were attacked, they kept walking or stopped, stunned, generally holding the leaf between their mandibles. In the case of being stunned, 5–15 ants of the same size or smaller approached the ant attacked and touched her with their antennae. This tending behavior towards the attacked ant could last for several minutes. Then, the attacked ant continued with the journey back to the nest. Usually, some workers stayed on the leaf carried by the attacked ant as hitchhikers. One *E. inferna* was captured by a hitchhiker. If a non-attacked ant nearby detected a fly, she tried to catch it with her mandibles. These ants opened their mandibles and raised their heads, adopting a biting posture once they detected the fly.

***Eibesfeldtphora trilobata*** (observations made over *At. vollenweideri* in San Cristóbal, Santa Fe province; Mercedes, Corrientes province; Argentina). These phorids perched at the side of the foraging trail on a leaf or stick, or even on the ground. They began flying after an ant walked under or near their perch. They followed the ant from behind for 5–10 sec (c. 2 m of the foraging trail), keeping a short distance. If by that time they did not reach the ant, they generally returned to the same perch or one near it, or, less frequently, they searched at flight for another close ant. They attacked on the neck, between the head and the thorax, probably on the foramen magnum, positioning their body in the same direction as the ant's body in a way that both heads were in the same direction. They attacked ants with or without loads, walking in both directions on the foraging trails.

The attacked ant stopped walking at the moment that it perceived the phorid, and when the phorid finished the attack, the ant adopted a C or ball posture, and maintained it for almost a minute before resuming its march. Some phorids of this species re-attacked the same individual ants after the ants abandoned the posture adopted in the previous attack. Some ants kicked the phorid flying near them using their hind legs. Others tried to dislodge the phorid by touching it with their legs and antennae. Some ants lay down on their side on the ground, and stayed there for some seconds; others started to run or did not show any reaction to the phorids. Around the attacked ant, many small ants adopted the biting or C postures. Several ants in biting postures were frequently observed near the area where the phorid perched. These flies seemed to be quite territorial with their perching site, as they kept the same perch or site in the foraging trail during the whole observation period.

***Lucianaphora*** folgaraitae Disney (observations made over *Ac. crassispinus, Ac. heyeri*, and *Ac. lundii* in Noetinger, Córdoba province, Argentina). This phorid went from perch to perch with short flying bouts, like jumping, and walked between the ants on the foraging trails, seemingly undetected by the ants. Phorids landed or walked on the leaves transported by the ants, and then moved to the ventral part of the head to put the ovipositor near the maxillae. It took these phorids 8–11 sec to oviposit.

If the ants detected the phorid, they stayed still or put their heads down towards the thorax, touching with their forelegs the ventral part of the head. In general, these reactions were effective to dissuade the phorid.

***Myrmosicarius brandaoi*** (observations made over *At. saltensis* in La María-INTA, Santiago del Estero, and *At. vollenweideri* in San Cristóbal, Santa Fe; P.N. Estero Poí, Formosa; R.N. Formosa, Formosa; Argentina). Most of the time, this phorid was flying along the foraging trail without following a particular ant. To attack, it touched the right side (14 times vs. 1 in left side) of the ant's head very fast (< 2 sec). The attacked ants could be carrying a leaf piece or not, and going in or out of the nest. This species was also collected flying inside the nest entrance hole.

The attacked ants adopted the ball posture, the C posture, or stayed in biting posture. Some ants adopted the biting or C postures without being touched by the phorid passing by. In several occasions, colony level response was evident by several small ants with the biting or C postures around the attacked ant. Those that were closer to the attacked ant could touch it with their antennae. One phorid of this species was captured by the ants.

***Myrmosicarius catharinensis*** (observations made over *Ac. ambiguus, Ac. heyeri* and *Ac. lundii* mostly in Magdalena, Buenos Aires province; San Cristóbal, Santa Fe province and Noetinger, Córdoba province, Argentina). This species flew along the foraging trail for great distances and speed without stopping. For example, a female covered 2 m in 30 seconds, and another 2 m in 25 seconds. In some instances, this species attacked ants performing nest maintenance, i.e., arranging sticks in the nest mound. Phorids of this species were able to fly sideways and backwards, maybe to locate or visually evaluate the ant. To attack, *M. catharinensis* went to the front of the ant and then darted to the ant's head, near the clypeus or the insertion of the antenna. It was frequent for this species to attack ants with their mandibles busy, carrying a load or clearing out sticks or soil particles from the trail, although it also attacked ants without loads.

The ants were able to detect the phorid while it was flying, and responded by either running or halting their march, disrupting ant traffic. While being attacked, ants adopted a ball or C posture. After the phorid attack, nestmates sometimes touched with their legs and antennae the area where the ant was attacked. In a few occasions, the ants showed no reaction to the phorid.

***Myrmosicarius cristobalensis*** (observations made over *Ac. fracticornis* and *Ac. lobicornis* mostly in San Cristóbal, Santa Fe province, and Noetinger, Córdoba province, Argentina). This species flew along the foraging trails, following ants that were (23 times) or not (8 times) carrying a leaf fragment, and following them very close to the gaster. For instance, one phorid followed an ant for 10 seconds at a 3-4 mm distance from the gaster. They were able to follow ants for 30 cm (circa 8 seconds), flying sideways or backwards. They attacked at the tip of the gaster, landing there for some seconds, with their head positioned in the same direction of the ant's head. During summer time, when ant foraging activity was reduced to a short time-window during the day, this species was attacking ants at refuse piles on a few occasions (11%), coincident with times when foragers were not active.

The attacked ants adopted C, ball, or biting postures; they halted for a few seconds or ran away. They also lowered the gaster against the ground, and some continued walking with the gaster between the hind legs. In one case, a phorid was chasing an ant from behind, and a nearby ant captured the phorid with its mandibles.

***Myrmosicarius crudelis*** (observations made over *Ac. crassispinus* and *Ac. lundii*, in San Cristóbal, Santa Fe and Noetinger, Córdoba, Argentina). These phorids showed several peculiarities. First, they were ovipositing in ants mainly at refuse piles, although they also searched for ants in foraging trails, always near the nest. They flew in a distinctive way, selecting one ant in which to oviposit while hovering in the air. They were also able to fly sideways and backwards, and go for a short distance into the entrance holes of the nest to attack ants. They attacked ants on the tip of the gaster, positioning their body in the same line as the ant's body, but with the ovipositor towards the left side of the ant. They attack ants loaded or not with the exhausted fungal substrate.

The attacked ant adopted a ball posture or lowered down the gaster while running back to the nest. In addition, the ants showed the biting posture when the phorids flew over them.

***Myrmosicairus gonzalezae*** (observations made of *At. vollenweideri*, in San Cristóbal, Santa Fe; and Mercedes, Corrientes, Argentina). This phorid flew fast along the foraging trail (for example, in one case a female moved 15 m in 30 seconds). This species oviposited between the head and thorax, in the right side of the host, with its body perpendicular to that of the ant. However, due to the posture of its body and the curved shape of its ovipositor (Disney et al. 2006), it is highly likely that the ovipositor tip reached the ventral side of the neck. *M. gonzalezae* mainly attacked ants not carrying a leaf (31 times) that were going out of the nest to forage, but it could also attack ants carrying a leaf (5 times). When flying, it followed the ants from behind.

The attacked ants stopped and touched with their legs and antennae the part of their bodies where the phorid oviposited. Other ants came and touched the attacked ant with their mouthparts and antennae. These ants adopted a C or biting posture as a colony response. After circa 15 seconds, the attacked ant did one of several things: started walking with the phorid still attacking her, turned themselves to one side against the ground, adopted a ball posture, or did not show any reaction at all. One phorid was captured by the ants.

***Myrmosicarius gracilipes*** (observations made over *Ac. crassispinus* in Noetinger, Córdoba province, Argentina). This phorid attacked ants at refuse piles, although it was also collected on foraging trails. The attacked ants were carrying a piece of exhausted fungal substrate or not, and the phorid attacked them on the right side, where the head articulates with the thorax, positioning its body perpendicular to the ant's body. The attacked ants adopted a biting posture when phorids flew over them.

***Myrmosicarius longipalpis*** (observations made over *Ac. hispidus* in San Cristóóbal, Santa Fe province; P.N. Chaco, Chaco province; P.N. Estero Poí, Formosa province, Argentina). This species searched for ants at the refuse pile. They followed ants very close to the host's front, flying backwards. They very quickly attacked the head of ants with refuse loads. In addition, they were able to search for an ant in which to oviposit from a fixed point in the air, from where they darted to the selected ant, in a similar way as *M. crudelis*. Usually, several females were attacking ants at a refuse pile, where it was frequently observed that two phorids touched in the air, and then separated to opposite sides of the refuse pile.

The attacked ant adopted biting or ball postures, and other ants approached the attacked ant, touching it with their mouth parts and antennae. When *M. longipalpis* phorids were flying, several ants adopted the biting posture.
